# A genome blogger manifesto

**DOI:** 10.1186/2047-217X-1-15

**Published:** 2012-10-26

**Authors:** Manuel Corpas

**Affiliations:** 1 Independent, Cambridge, UK

**Keywords:** Genome, Blogging, Personal genomics

## Abstract

Cheap prices for genomic testing have revolutionized consumers’ access to personal genomics. Exploration of personal genomes poses significant challenges for customers wishing to learn beyond provider customer reports. A vibrant community has spontaneously appeared blogging experiences and data as a way to learn about their personal genomes. No set of values has publicly been described to date encapsulating ideals and code of conduct for this community. Here I present a first attempt to address this vacuum based on my own personal experiences as genome blogger.

## Background

Genome blogging refers to the sharing of experiences, results and data relative to a person’s genome. Normally, the information shared belongs to the blogger him/herself, but at times it belongs to other people (related or not) who provide their explicit consent to share personal data and views. Genome blogging has established itself in recent years as a vibrant online community, particularly after the availability of affordable direct-to-consumer (DTC) personal genomic tests. A number of public resources have been developed embracing a spirit of openness when sharing or discussing personal genome data. The Personal Genome Project (PGP) [1] has been a pioneering initiative, sharing genomes for research in a clinical setting. SNPedia [2] is another important resource supporting personal genome annotation. More recently, openSNP [3] has been developed as a central repository of personal DNA genomics with inbuilt capabilities for annotation.

Reasons why people may want to share personal genomic information vary. These include a) philanthropic reasons, b) interest in discussing specific results, and c) exposing the data freely to new potential analysts. This latter motivation justifies genome sharing, considering that no DTC report encompasses all available knowledge present in the scientific literature. It still remains unknown however, the extent to which genome sharing may negatively affect an individual. There is a perceived risk that it may engender genetic discrimination, loss of personal privacy or even identity theft.

Although no formal document has been produced to date on the core values inspiring personal genome sharing through blogging, a set of consensus rules driving it could be made explicit. Here I present a first attempt in writing a genome blogger code of conduct. These are not fixed values, on the contrary, I expect these to develop as the debate evolves. I base some of the ideas below on Marcus Wohlsen’s ‘Biopunk’ book [4], Meredith Patterson’s ‘biopunk manifesto’ [4], Misha Angrist’s 'Here is a human being' [5] and Pekka Himanen's ‘Hacker's ethics’ book [6].

### Main Text

1. **Intelligent exploration**, **experimentation and trial to push the boundaries of knowledge are a right for ordinary people**. The days in which genetic science was only done by university professors or people working in corporate labs are now over. Everyone should have the power and legitimacy to be able to discover, develop and find new things about their own genome data.

2. **Sharing can be more useful than keeping data to oneself**. Whether one wants to share genome data or keep it private should be a matter of personal choice. The data encoded in my genome are mine to measure, use and distribute as I please, without restriction. Sharing personal experiences about personal genomics findings is a way of creative self-expression and a channel for curiosity to find its way to better self-awareness.

3. **Whatever attitude a person has towards personal genome privacy**, **it should be utterly respected**. Knowledge of genetics is not as important for informed consent as the personal/psychological attitude of the person. The decision of openness towards personal genome data completely resides in the individual and although openness is the better default option, privacy must be utterly respected and not judged.

4. **Personal genome data access should be affordable to all irrespective of nationality**, **gender**, **social background or any other circumstance**. Access to personal genomics data and tools for its interpretation should become accessible to everyone, not just the realm of those who can pay big sums of money for it. Not having access to a personal genetic test is in itself a new kind of discrimination.

5. **Stating that genetic tests merely provide non**-**clinical information misses the point of what personal genomics is all about**. Most genomic information is uninterpretable and may well be meaningless. But those are not reasons to deny it to people. Genetic risks tests do tell something about one’s health, one’s ability to respond to certain drugs and one’s ethnic ancestry.

6. **Genes affect one**’**s present but they do not determine one**’**s future**. One’s genetic data is just one more factor among many others in predispositions, risks and behavioral reactions. Genes are affected by the environment in the same way as the environment is affected by genes. Although some dramatic genetic diseases can significantly alter one’s life style, genes can never determine the decisions that make us who we are.

7. **Education in risks and opportunities for personal genetic testing should be the primary aim of policy makers**. Restricting access to interested people makes no sense and it is virtually impossible to ensure. The power of the Internet allows anyone to order a test with no need to physically be in any particular location.

8. **Corporate interest can never compromise any human right**. Laws must fully protect individual human rights of equality for every person, irrespective of predicted risks from genetic data.

9. **Privacy does not have to be incompatible with openness**. Knowing one’s bank account movements or shopping habits are probably a better predictor of one’s personality than a personal genome.

## Discussion

For the foreseeable future, the challenge of personal genomics will continue to lie in the interpretation aspect. Genome blogging offers a way of sharing experiences and raising self-awareness. Sharing one’s experiences on the Internet may help start answering individualized questions, but the personal genomics field will continue to be inaccessible to most people unless more openness with data and tools is nurtured. More availability of tests does not guarantee better access to personal genomic data; it may overwhelm consumers. Promoting recognition of rights for ordinary citizens who wish to share or keep private personal genomics experiences is a fundamental pillar underlying genome blogging community values. Genome blogging will take off if/when genomic tests are used by a significant fraction of the population. I believe genome blogging has a role to play in the personalized medicine of the future.

## Abbreviation

DTC: Direct-to-consumer.

## Competing interests

The author declares he has no competing interests.

## Authors’ contributions

MC conceived and wrote the paper.
